# Hydrochar Produced from Orange Peel Essential Oil Extraction Residues as a Support for Nanoparticles in Hydrogen Evolution Catalysis from NaBH_4_

**DOI:** 10.1021/acsomega.6c01972

**Published:** 2026-07-22

**Authors:** Nelson Jonathan, Renata P. L. Moreira, Noemí C. S. de Souza, Amanda C. Filgueiras, Garbas A. dos S. Junior, Patrícia F. Pinheiro

**Affiliations:** † Department of Chemistry, 702064 Adamawa State College of Education, Hong, Hong, Adamawa State 640102, Nigeria; ‡ Departamento de Química, 28120 Universidade Federal de Viçosa, Av. Peter Henry Rolfs, s/n Campus Universitário, Viçosa, Minas Gerais CEP 36570-900, Brazil

## Abstract

This work explores the valorization of orange peel residues from essential oil extraction as support for Co/Pt nanoparticles in the catalytic hydrolysis of sodium borohydride (NaBH_4_) for hydrogen production. Hydrochar was produced via hydrothermal carbonization at 130 °C for 6 h, preserving surface functional groups that facilitate metal nanoparticle dispersion. Co/Pt nanoparticles were successfully deposited on the hydrochar, as confirmed by TEM, SEM, EDS, and XRD analyses, revealing homogeneous distribution and nanoscale size. FTIR, TGA and BET characterization demonstrated a chemically rich, mesoporous structure, conducive to high catalytic performance. The catalytic activity was evaluated through NaBH_4_ hydrolysis under varying metal loadings, NaOH concentrations, NaBH_4_ concentrations, and temperatures. Monometallic Pt and Co achieved hydrogen generation rates (HGR) of 2573 and 4074 mL·min^–1^·g^–1^, respectively, while a 50:50 Co/Pt bimetallic composition showed a synergistic effect, reaching 3359 mL·min^–1^·g^–1^. Durability assays indicated that the catalyst retained approximately 95% of its initial activity over ten consecutive cycles. Kinetic isotope effect (KIE) experiments using H_2_O and D_2_O revealed a high KIE value of 4.97, indicating that water activation is the rate-determining step. This work demonstrates that orange peel residues can be efficiently repurposed as a sustainable catalyst support, combining waste valorization with environmentally friendly hydrogen production. The approach offers a cost-effective and green strategy for generating hydrogen while contributing to circular economy initiatives.

## Graphical abstract

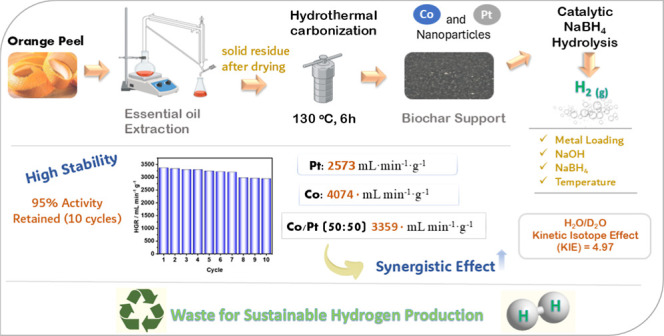

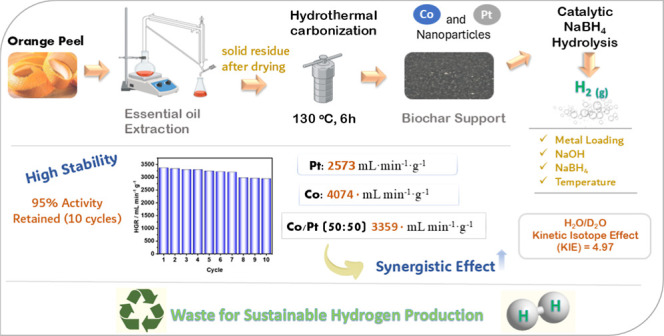

## 1 Introduction

The global essential oils market was valued at approximately USD 13.66 billion in 2025 and is projected to grow at a compound annual growth rate (CAGR) of 11.08% through 2034, driven by increasing demand in wellness, personal care, and natural products sectors.^1^ Among the most widely traded products, orange oil ranks first, accounting for approximately 22% of the market’s total revenue.^2^ Extracted mainly from the fruit peel, this oil contains bioactive compounds that have attracted increasing interest and are widely used in food, cosmetic, and pharmaceutical industries. In addition to essential oil, the production of high value-added bioproducts from orange residues has been gaining prominence and is becoming an increasingly common practice in countries that cultivate the fruit on a large scale.^3,4^

During extraction processes used for essential oil production, such as the distillation of plant biomass, a substantial amount of byproducts is generated, including wastewater and exhausted solid material.^5^ Although often overlooked, these residues may account for most of the initial biomass and still contain valuable compounds, offering considerable potential for recovery and reuse within a circular economy framework. One promising valorization route involves the production of biochar-carbon-rich materials characterized by high specific surface area and diverse functional groups, which enable their application across a wide range of environmental, agricultural, and industrial processes.^6–8^

There are two main routes for producing biochar: pyrolysis and hydrothermal carbonization.^9^ The hydrothermal process offers the advantage of operating under milder temperature conditions, which allows for better preservation of surface functional groups. In this method, reactions occur in water under subcritical conditions, at pressures high enough to maintain the liquid phase.^10^ Under these conditions, water exhibits unique properties, such as a lower dielectric constant, increased diffusion coefficient, and the ability to act simultaneously as a solvent and a reactant, thereby facilitating controlled chemical transformations and promoting higher heteroatom retention in the resulting biochar. Among the various applications of biochars, their use as catalysts in hydrogen (H_2_) release processes from chemical hydrogen storage materials is particularly noteworthy.^11,12^

H_2_ is widely regarded as a key fuel of the future.^13^ The burning of fossil fuels remains the primary source of anthropogenic emissions, and the byproducts generated during their combustion pose severe risks to ecosystems.^14^ The ongoing depletion of global oil reserves and the intensification of environmental challenges have made energy and environmental concerns critical constraints on development worldwide. In this context, clean and renewable energy sources have gained increasing attention.^15,16^

Hydrogen stands out as a particularly promising energy carrier due to its nonpolluting nature and high efficiency in energy conversion processes. It has a calorific value three times higher per unit mass (142 MJ·kg^–1^) than gasoline and generates only water vapor upon use.^17,18^ Owing to these advantages, hydrogen has become a central component of strategies to mitigate global warming, especially when produced through carbon-neutral pathways. However, its practical use is hindered by challenges related to storage and transportation, as hydrogen is highly volatile, exhibits extremely low melting and boiling points, and is prone to explosive behavior under certain conditions.^19^

There are several routes for hydrogen production, and among them, metal-hydride hydrolysis offers notable advantages due to the ease of handling these solid materials, which also facilitate storage and transportation.^20^ A prominent example is sodium borohydride (NaBH_4_), whose inherently slow hydrolysis rate can be significantly enhanced using acidic catalysts, leading to the formation of sodium metaborate (NaBO_2_), according to eq 1. This byproduct is nonpolluting and can be fully recycled, making the process particularly attractive for sustainable hydrogen generation.^21,22^

1 $${NaBH}_{4}+{2H}_{2}O\to {NaBO}_{2}+{4H}_{2}↑$$

NaBH_4_ is a hydrogen transfer agent with the potential to store up to approximately 10.8% hydrogen by weight.^23^ Additionally, its safe storage and the rapid release of hydrogen gas upon contact with water make it a viable option for storing and transporting hydrogen in solid form. This eliminates the need for heavy high-pressure gas tanks and pipelines.^24^

In addition to NaBH_4_, other amine–borane complexes, including ammonia borane and ethylenediamine bisborane, have been extensively investigated as promising hydrogen storage materials, particularly in catalytic hydrolysis systems employing heterogeneous carbon-based catalysts.^25,26^

This work demonstrates, for the first time, that residues from orange peel essential oil production can serve as a support for Pt and Co nanoparticles in the catalytic hydrolysis of sodium borohydride (NaBH_4_) for hydrogen generation. By valorizing this previously unexploited waste stream, the approach addresses waste management challenges while enabling a cost-effective and sustainable catalyst for environmentally friendly hydrogen production.

## 2 Experimental Section

### 2.1 Materials

All reagents were of analytical grade. The hydrochloric acid (37%, CAS7647-01-0), used in the synthesis of hydrochar, was purchased from Química Moderna and is of analytical grade. Sodium borohydride (98%, CAS 16940-66-2) and cobalt nitrate hexahydrate (98%, CAS 10026-22-9) were supplied by Vetec, while hexachloroplatinic acid hexahydrate (99.9%, CAS 18497-13-7) was obtained from Sigma-Aldrich. Solutions were prepared using ultrapure Type I water from a Milli-Q system (Merck Millipore).

### 2.2 Biomass Collection and Pretreatment of Orange Peel Residue

Fresh orange-peel waste was collected from a local market in Viçosa, Minas Gerais, Brazil, and transported to the Laboratory for Analysis and Synthesis of Bioactive Organic Molecules (LAMOB) at the Universidade Federal de Viçosa (UFV). The orange-peel waste (OPW) was first blended, and the essential oil was extracted via steam-distillation. The resulting orange peel residue (OPR) was dried at 80 °C for 3 days, then ground into fine particles. To promote demineralization and remove residual impurities, the OPR was treated in a dilute hydrochloric acid (HCl) solution (OPR/HCl) for 72 h (OPR/HCl 1:3­(w/v)). The material was subsequently washed several times with ultrapure water until neutral pH was reached, oven-dried, and ground again to obtain a fine powder suitable for further applications (Scheme 1).

**Scheme 1:**
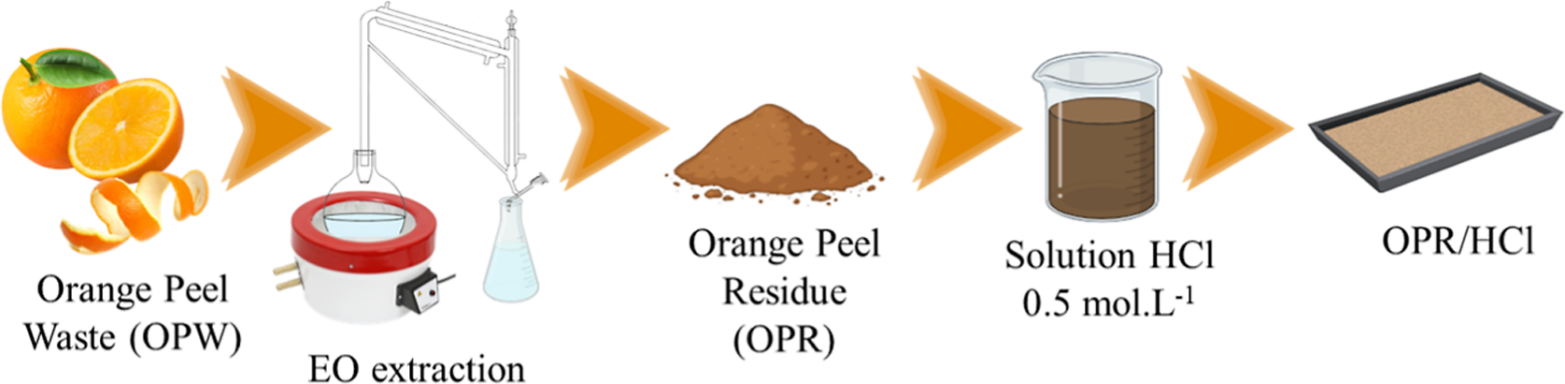
Illustrative Diagram of the Steps for Pre-Treatment of Orange Peel Residue

### 2.3 Hydrochar Production

One gram of OPR was placed in a tube, and 10 mL of distilled water was added. The mixture was stirred for 30 min using a magnetic stirrer, then transferred to an autoclave reactor. Hydrothermal partial carbonization of the OPR was performed in the autoclave at 130 °C for 6 h. After the reaction, the material was washed with distilled water until neutral pH was reached, oven-dried, and ground into a fine powder, which was designated as Orange Peel hydrochar (OPHC) (Scheme 2).

**Scheme 2:**
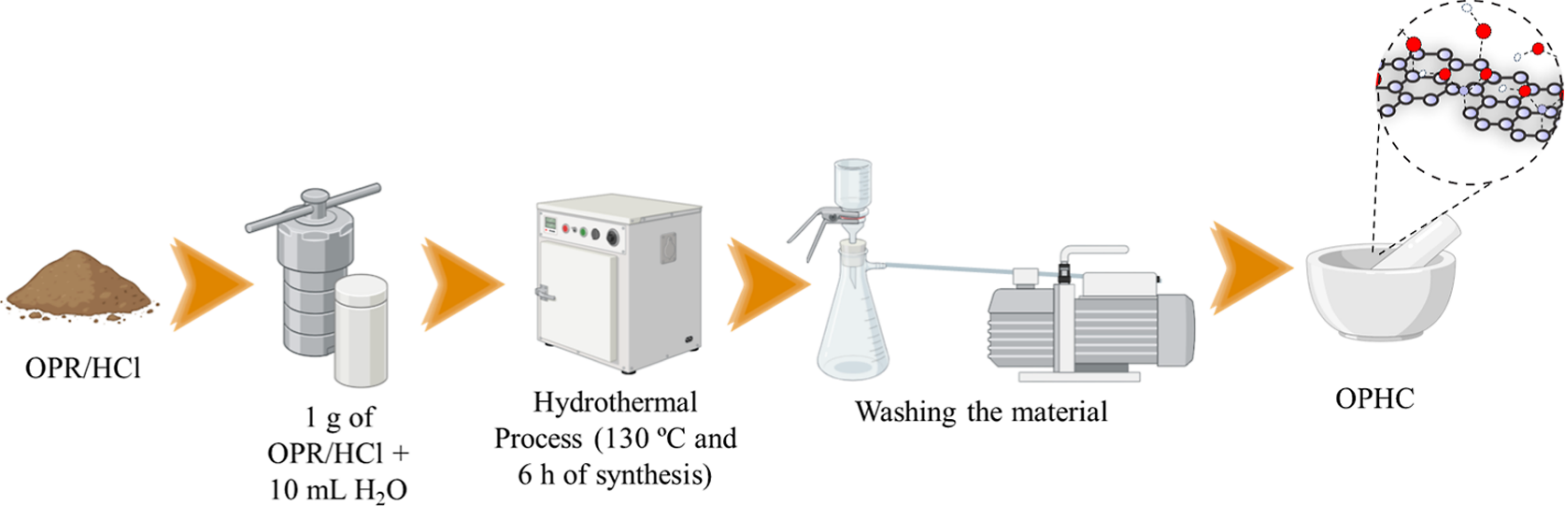
Illustrative Diagram of the Steps in the Synthesis of Orange Peel Hydrochar

### 2.4 Synthesis of Co/Pt Nanoparticles Decorated on Hydrochar

The Co/Pt nanoparticles catalysts supported on hydrochar were synthesized using metal precursors in varying percentages, following the method described by Sperandio et al.^27^ (as shown in Table 1). Ten milligrams of hydrochar were placed in a beaker, and 10 mL of distilled water was added. The mixture was stirred for 10 min using a magnetic stirrer. The Pt and Co precursor salts were weighed according to the proportions indicated in Table 1 and added to the solution, which was stirred for an additional 10 min. Subsequently, 40 mg of sodium borohydride, dissolved in 1.0 mL of water, was added dropwise under stirring over 10 min until the reduction reaction was complete. The resulting mixture was centrifugated at 4000 rpm for 10 min. The resulting solid was washed with ultrapure water followed by centrifugation (4000 rpm for 10 min), and this process was repeated three times (Scheme 3).

**Table 1:** Composition of Co/Pt Nanoparticles Decorated on Hydrochar Derived from Essential Oil Residues

platinum		cobalt	
%	*n* (mmol)	%	*n* (mmol)
100	0.0250	0	0
80	0.0200	20	0.0050
60	0.0150	40	0.0100
50	0.0125	50	0.0125
40	0.0100	60	0.0150
20	0.0050	80	0.0200
0	0	100	0.0250

**Scheme 3:**
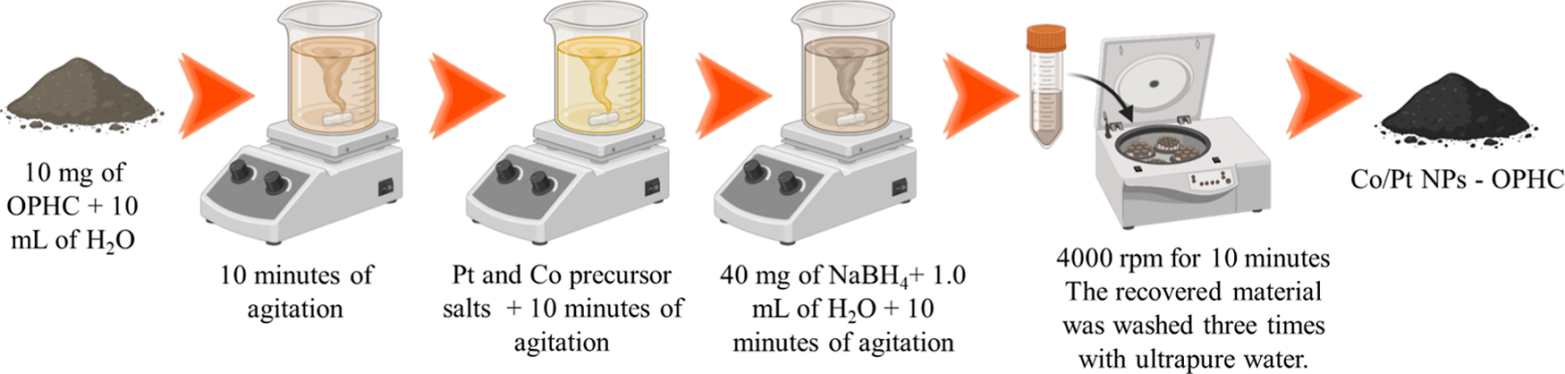
Illustrative Diagram of the Steps of Synthesis of Co/Pt Nanoparticles Decorated on Hydrochar

### 2.5 Characterization of Materials

The carbon, hydrogen, nitrogen, and sulfur contents were measured by elemental analysis using a LECO TruSpec Micro analyzer, and the oxygen content was estimated by difference, subtracting the combined carbon (C), hydrogen (H), nitrogen (N), and sulfur (S) percentages from 100%.

Fourier-transform infrared (FTIR) spectra were obtained using a Bruker ALPHA II spectrophotometer with an attenuated total reflectance (ATR) accessory in the range of 400–4000 cm^–1^, using 16 scans at 4 cm^–1^ resolution.

X-ray diffraction (XRD) patterns were recorded on a Bruker D8-Discover diffractometer using Cu–Kα radiation (λ = 0.1541 nm) over a 2θ range of 10°–70°.

Transmission Electron Microscopy (TEM) was performed on a Tecnai G2–20 SuperTwin FEI 200 kV microscope, coupled with a Si–Li detector (EDAX) for Energy Dispersive X-ray Spectroscopy (EDS).

The surface morphology was analyzed by scanning electron microscopy (SEM, JSM-6010LA, JEOL) following gold sputter coating (Quorum Q150R S). Elemental composition was assessed using SEM coupled with an energy-dispersive X-ray spectroscopy (EDS) system equipped with a silicon detector, with a resolution of 133 eV.

Thermal properties were evaluated using a Shimadzu DTG60H thermobalance under a nitrogen atmosphere (50 mL min^–1^) over 30–800 °C. Simultaneous thermogravimetric (TG) and differential thermal analysis (DTA) data were collected, with a heating rate of 10 °C min^–1^. TG curves were differentiated (DTG) to confirm thermal events.

Nitrogen adsorption–desorption isotherms were measured on a Nova 600 Series instrument (Anton Paar). Samples were degassed at 120 °C for 4 h prior to analysis, and the Brunauer–Emmett–Teller (BET) method was applied to calculate the surface area.

### 2.6 Hydrolysis of Sodium Borohydride for Hydrogen Evolution

The experiments were conducted following the procedure described by Silva de Souza et al.^28^ Ten milligrams of the freshly synthesized Co/Pt NPs-hydrochar catalyst were dispersed in 2 mL of Milli-Q water and added to the reaction setup. The system consisted of a Schlenk tube flask connected to a water-filled buret, where the hydrogen gas produced displaced the water column, which was recorded throughout the reaction. After assembling the system, 1.00 mL of NaBH_4_ solution (0.500 mol L^–1^) was injected through a septum. The reaction was carried out under constant stirring, ambient pressure (0.93 atm), and controlled temperature.

The volume of hydrogen generated was measured by water displacement in the buret. The pressure (*P*) exerted by the evolved hydrogen within the liquid column was calculated using eq 2, taking into account the following parameters: water density (ρ) = 1000 kg m^–3^, gravitational acceleration (*g*) = 9.78 m s^–2^, local atmospheric pressure = 942,589 Pa (707 mmHg), and the observed water displacement (*h*) in meters.

2 $$P=P_{0}+\rho gh$$

The hydrogen generation rate (HGR) according to eq 3.

3 $$HGR=\frac{{\Delta V}_{H_{2}}}{\Delta t\times m_{cat}}$$

where ΔV_H_2__ is the change in hydrogen volume (mL), Δ*t* is the reaction time (min), and *m*_cat_ is the mass of catalyst (g).

Different reaction conditions were investigated, as detailed below.

#### 2.6.1 Variation of Metal Loading and Bimetallic Composition

Different loadings of Co/Pt bimetallic nanoparticles supported (2.0, 4.0, 5.0, and 6.0 mmol % relative to NaBH_4_) on hydrochar (10 mg) were evaluated. All other parameters were kept constant, including the hydrochar amount (10 mg), NaBH_4_ solution (1.00 mL, 0.500 mol L^–1^, corresponding to 0.5 mmol), and temperature (25 °C). Accordingly, 0.01, 0.02, and 0.03 mmol of the bimetallic composition were used, respectively.

#### 2.6.2 Variation of Sodium Hydroxide Concentration

NaOH concentrations of 0.010, 0.025, 0.050, and 0.075 mmol L^–1^ were evaluated. For these experiments, 0.50 mmol of NaBH_4_ was dissolved in 1.00 mL of NaOH solution at the specified concentration before injection. All other parameters were kept constant (Co/Pt NPs: 0.025 mmol supported on 10 mg of hydrochar; temperature: 25 °C). The NaOH concentrations were intentionally kept low to avoid changes in the reaction medium properties, particularly viscosity, which could interfere with mass transfer and catalytic performance.

#### 2.6.3 Variation of Sodium Borohydride Concentration

The effect of NaBH_4_ was evaluated by varying its amount (0.3, 0.4, 0.5, and 0.6 mmol), corresponding to concentrations of 0.3, 0.4, 0.5, and 0.6 mol L^–1^ (total volume: 1.00 mL). The other parameters were kept constant: Co/Pt NPs: 0.025 mmol supported on 10 mg of hydrochar and temperature (25 °C).

#### 2.6.4 Effect of Reaction Temperature

Hydrogen evolution was evaluated at different temperatures: 20 °C, 30 °C, and 40 °C, keeping other parameters constant (Co/Pt NPs: 0.025 mmol supported on 10 mg of hydrochar; temperature: 25 °C), NaBH_4_ 1.00 mL of 0.500 mol L^–1^ solution).

#### 2.6.5 Durability

After each catalyst–use cycle, the Schlenk tube was disconnected only to reset the buret before beginning the next cycle. For every repetition, 1.00 mL of fresh NaBH_4_ solution (0.500 mol L^–1^) was added without washing the catalyst and without removing it from the Schlenk tube. The following parameters were kept constant: Co/Pt NPs: 0.025 mmol supported on 10 mg of hydrochar; temperature: 25 °C. Durability was assessed over ten consecutive cycles performed under identical conditions.

#### 2.6.6 Evaluation of the Kinetic Isotope Effect (KIE)

To investigate the catalytic reaction mechanism, the catalyst previously washed with isopropanol and dried under vacuum to remove any residual water, was placed into a Schlenk tube containing 1.0 mL of deuterium oxide (D_2_O). The system was sealed with a septum and connected to a water-filled buret. Then, 1.00 mL of fresh NaBH_4_ solution (0.500 mol L^–1^) prepared in D_2_O was injected into the system. All reactions were conducted at 25 °C. The KIE was calculated according to eq 4.

4 $$KIE=\frac{k_{H}}{k_{D}}$$

where *k*_H_ and *k*_D_ correspond to the rate constants in H_2_O and D_2_O (deuterated water), respectively.

## 3 Results and Discussion

In this work, materials were synthesized from orange peel residues after essential oil extraction, converted into hydrochar and modified with Co/Pt nanoparticles for hydrogen generation from sodium borohydride. The hydrothermal treatment at 130 °C was selected because subcritical water enhances biomass conversion while preserving functional groups important for catalyst support applications.^27–32^

### 3.1 Characterization

Initially, the OPHC was characterized by elemental analysis. The material contained approximately 47.4 wt % C, 6.1 wt % H, and ∼1.4 wt % N, likely due to residual proteins or amino compounds and S was not detected. The oxygen content was estimated by difference to be around 46 wt %, reflecting the presence of oxygenated functional groups on the hydrochar surface. The orange peel residue-derived hydrochar exhibited atomic ratios of H/C ∼1.52 and O/C ∼0.74, indicating a partially carbonized structure with significant oxygen- and hydrogen-containing functional groups. The relatively high H/C ratio suggests limited aromatization, while the O/C ratio reflects the presence of surface oxygen functionalities, which may contribute to the material’s chemical reactivity.^30,33,34^

The FTIR spectrum of OPR/HCl, OPHC, and Co/PtNPs-OPHC revealed several characteristic absorption bands (Figure 1a). A broad band around 3328 cm^–1^ corresponds to O–H stretching vibrations of hydroxyl groups, typically associated with alcohols and phenols. These groups enhance the surface hydrophilicity and polarity, promoting hydrogen bonding and facilitating adsorption of polar molecules.

**Figure 1:**
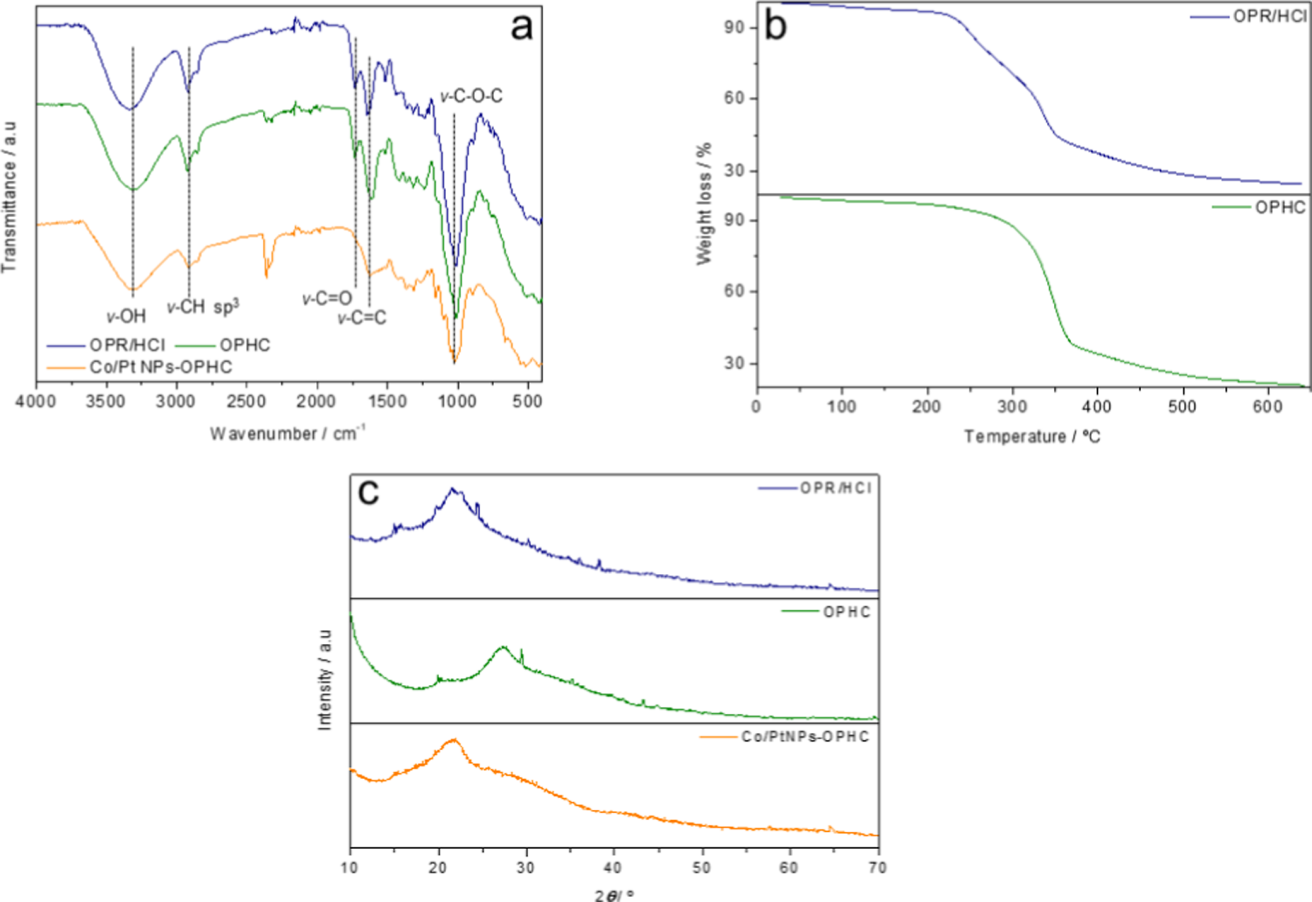
(a) Fourier-transform infrared (FTIR) spectrum, (b) thermogravimetric (TG) analysis, and (c) X-ray diffraction (XRD) pattern.

Bands at 2918 cm^–1^ are attributed to C–H stretching in aliphatic structures, indicating partial retention of organic functionalities after pyrolysis. Carbonyl (C=O) stretching vibrations near 1728 cm^–1^ suggest the presence of carboxylic acids, ketones, or aldehydes, which can assist in metal ion coordination and improve nanoparticle binding and dispersion. Aromatic C=C stretching bands around 1642 cm^–1^ indicate the formation of stable conjugated carbon domains, enhancing electrical conductivity and thermal stability, important features for electrocatalytic applications.^35^ Finally, bands near 1015 cm^–1^ are assigned to C–O–C ether groups or C–O stretching in esters, contributing further to surface polarity and reactivity. These observed bands are consistent with those reported in the literature.^7,30,34^

TGA (Figures 1b and S1a,b) revealed three distinct stages of decomposition for the OPR/HCl material and only two for OPHC, with approximately 20% residual carbon remaining at high temperatures for both. An initial low weight loss (∼2%) below 100 °C is attributed to the removal of adsorbed moisture, in agreement with observations reported by Netto et al.^34^ Subsequent thermal degradation of OPR/HCl occurs within well-defined temperature ranges: between 193 and 275 °C (∼20% mass loss) and between 301 and 370 °C (∼20% mass loss). These ranges are characteristic of hemicellulose and cellulose degradation in biomass, reflecting the gradual breakdown of the macromolecules that compose the biomass.^36,37^ For the OPHC support, the second stage of degradation occurred between 247 and 391 °C, with a mass loss of ∼60%, indicating that all macromolecules in the biomass were degraded in a single stage, as also evidenced by Filgueiras et al.^8^

The XRD pattern (Figure 1c) revealed that the carbon matrix is predominantly amorphous, evidenced by a broad peak between 20° and 30° 2θ, consistent with literature reports.^34^ The absence of sharp diffraction peaks for Pt or Co is noteworthy, suggesting that the nanoparticles are either highly dispersed on the hydrochar surface or encapsulated within the carbon layers.

The specific surface area (Figure 2a–d) determined by the BET method was 0.97 m^2^.g^–1^ for the orange peel residue (OPR), 0.00 m^2^.g^–1^ for orange peel residue (OPR) treated in dilute hydrochloric acid (HCl) solution (OPR/HCl), 0.40 m^2^.g^–1^ for the support (OPHC), and 2.67 m^2^.g^–1^ for the catalyst containing nanoparticles (Co/PtNPs-OPHC) (Table S1). The relatively low BET surface area values can be attributed to the nature of the precursor, as the orange peel residues were previously subjected to essential oil extraction. This process removes volatile compounds and may promote structural collapse or pore blockage during subsequent carbonization, resulting in materials with lower porosity. Additionally, the mild hydrothermal conditions employed also contribute to the limited development of porous structure. Similar behavior has been reported in the literature, where hydrochars derived from orange peel and bagasse also exhibited low specific surface areas.^29,38^ Despite the low surface area, the material still provides sufficient active sites for nanoparticle dispersion and catalytic performance.

**Figure 2:**
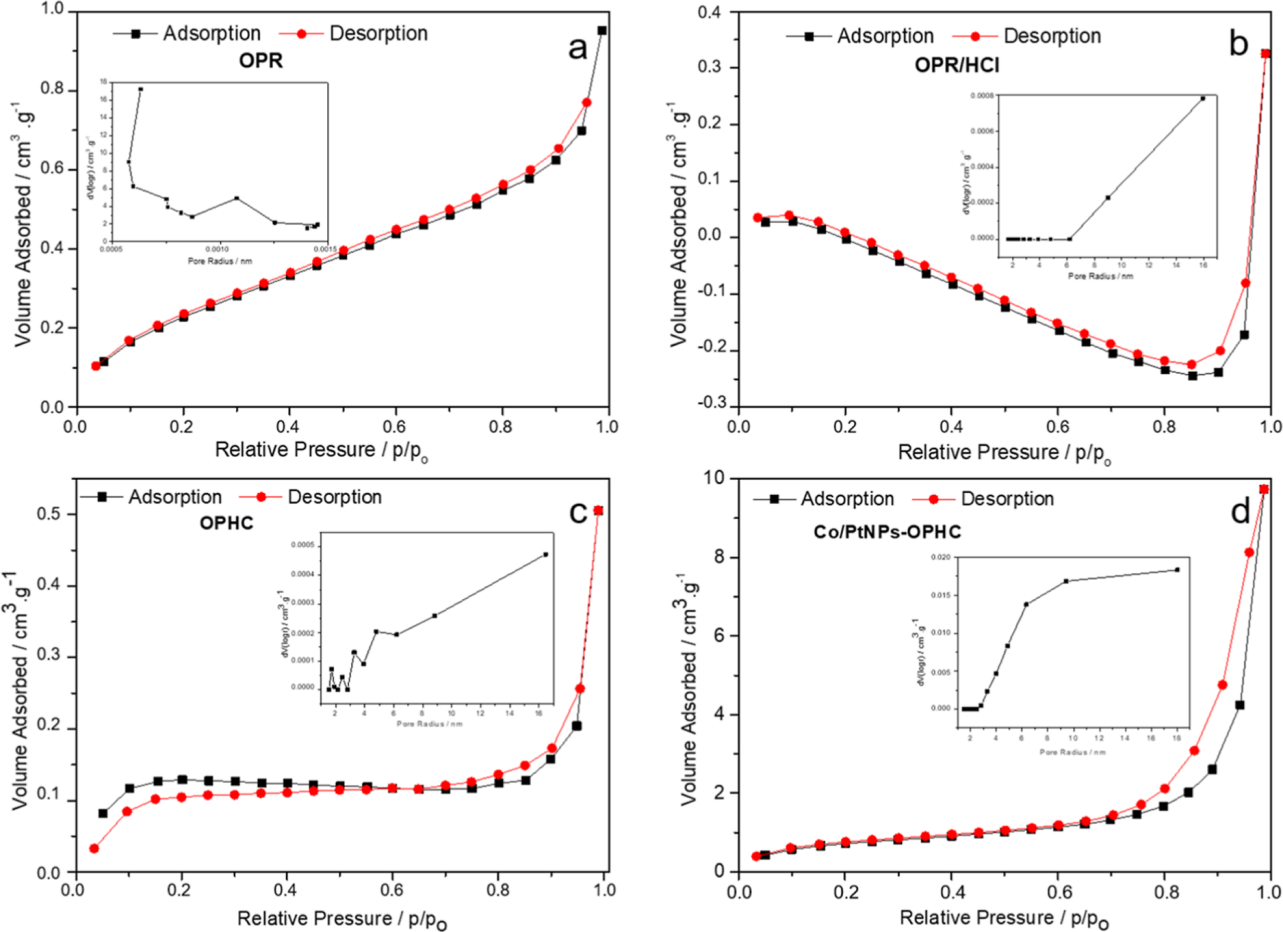
(a) Nitrogen adsorption–desorption isotherm of orange peel residue (OPR), (b) orange peel residue (OPR) was treated in a dilute hydrochloric acid (HCl) solution (OPR/HCl), (c) OPHC, and (d) Co/PtNPs-OPHC.

The decrease in surface area after HCl treatment (OPR/HCl) suggests that the acid treatment does not contribute to the formation of new surface area at this stage. Instead, this behavior is consistent with a structural rearrangement, which may involve pore widening or the removal of species that previously contributed to nitrogen adsorption measurements, without effectively increasing the accessible surface area.^39^

After hydrothermal carbonization (OPHC) and subsequent incorporation of metal nanoparticles (Co/PtNPs-OPHC), a slight increase in surface area is observed. This may be associated with structural reorganization during carbonization and/or the dispersion of nanoparticles, which can contribute to additional accessible surface or prevent complete pore collapse.

The pore size distribution obtained by the Barrett–Joyner–Halenda (BJH) method indicates that pores smaller than 2 nm are classified as micropores, while pores in the range of 2 to 50 nm are classified as mesopores, according to the IUPAC classification.^40^ All materials are classified as mesoporous, with diameters ranging from 3.08 to 31.92 (Table S1).

The materials were characterized by scanning electron microscopy (SEM). The starting materials (OPR and OPR/HCl) (Figure S2), as well as the resulting hydrochar, do not exhibit significant morphological changes. All samples present an irregular morphology without a well-defined structure, along with rough surfaces that are typically observed in raw biomass and hydrochar derived from lignocellulosic precursors.^8,34^ After the incorporation of the Co/Pt NPs, the presence of particles distributed across the material’s surface becomes evident, confirming the successful synthesis and deposition of the nanoparticles (Figure 3a,b).

**Figure 3:**
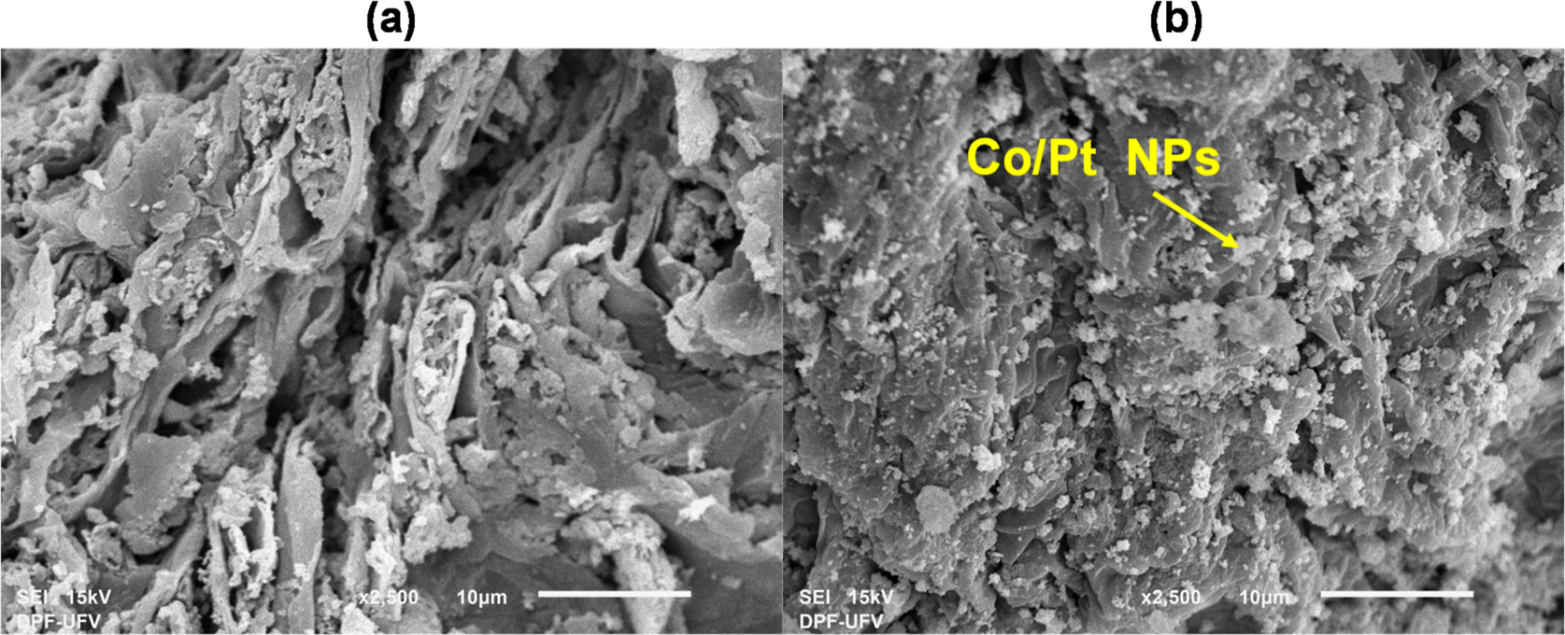
Scanning electron microscopy (SEM) images (a) orange peel residue-derived hydrochar (OPHC) and (b) Co/Pt nanoparticles decorated on OPHC (Co/Pt NPs-OPHC).

The EDS images shown in Figures S3 and S4 allow evaluating the elemental composition of the OPHC and of the material after the deposition of the Co/Pt NPs-OPHC. In the spectrum of the OPHC, characteristic signals of carbon, oxygen, sulfur, iron, magnesium, calcium, and sodium are observed, which are consistent with the typical composition of hydrochar. After functionalization with the nanoparticles, the appearance of Co and Pt becomes evident, confirming their incorporation into the matrix. It should be noted that the reported values are semiquantitative, serving mainly to indicate the presence of the elements and relative comparisons rather than to determine absolute concentrations.

The materials were characterized by TEM (Figure 4a–d). The images show nanoparticles (darker spots) homogeneously distributed over the carbon sheets (lighter regions).

**Figure 4:**
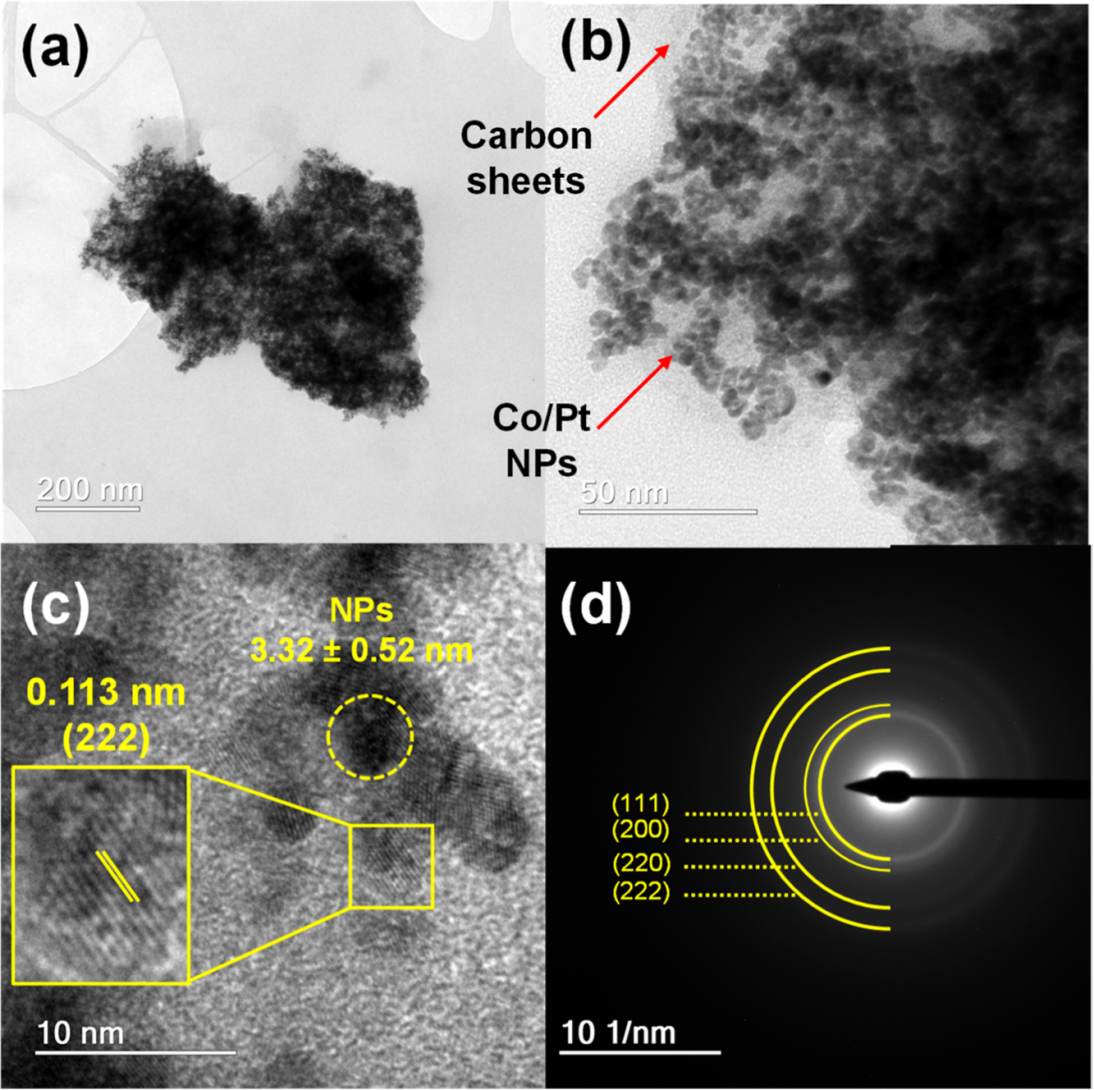
(a,b) TEM images of Co/Pt nanoparticles decorated on orange-peel-derived hydrochar (Co/Pt NPs-OPHC); (c) nanoparticle size distribution; (d) SAED diffraction pattern.

The nanoparticles exhibited an average diameter of 3.32 ± 0.52 nm, with an interplanar spacing of 0.222 nm. The SAED analysis revealed diffraction rings with interplanar spacings of 2.22 Å (111), 1.91 Å (200), 1.34 Å (220), and 1.13 Å (222). These values match the characteristic fcc diffraction planes reported in the JCPDS card 04-0802 for Pt^0^, indicating that the Pt component retains its metallic crystalline structure. The presence of these (hkl) planes confirms the formation of Co/Pt bimetallic nanoparticles, in which Pt crystallites dominate the diffraction pattern while Co is incorporated into the metallic domain.

### 3.2 Application

The synthesized material was applied to the hydrogen evolution reaction from NaBH_4_ (Figure 5). As shown in Figure 5a, monometallic Pt and Co nanoparticles exhibited HGR values of 1485 and 538 mL·min^–1^ g^–1^, respectively. After embedding into the orange-peel-derived hydrochar, the HGR increased to 2573 mL·min^–1^ g^–1^ (Pt) and 4074 mL·min^–1^ g^–1^ (Co). This enhancement is attributed to the catalytic properties of the hydrochar, whose chemically rich surface (FTIR, Figure 1a) promotes metal impregnation and provides multiple interaction sites. XRD (Figure 1b) confirms the good dispersion of metallic nanoparticles on the carbon matrix, a factor known to improve catalytic efficiency. The partially amorphous structure of hydrochar also contributes to defect sites, and the presence of many surface functional groups increases metal anchoring, corroborating its suitability as an efficient adsorbent and catalytic support, consistent with previous reports. The strong catalytic performance observed for the individual Co and Pt nanoparticles motivated the investigation of bimetallic compositions. To this end, Pt–Co composites were synthesized with different molar ratios to evaluate possible synergistic effects. Figure 5b shows that catalytic activity varies significantly with composition. Although the introduction of Co generally decreases the reaction rate compared to monometallic Pt, the 50:50 formulation exhibits the best performance, reaching the highest HGR (3359 mL min^–1^ g^–1^). As previously described, TEM and SAED analyses show homogeneously distributed nanoparticles with fcc diffraction planes corresponding to Pt^0^, and evidence that Co is incorporated into the metallic domain, supporting intimate contact and electronic interaction between Pt and Co. This behavior indicates that, at this ratio, there is an optimal balance of electronic and structural contributions from both metals. The 50:50 proportion enhances the electronic interaction between Pt and Co and promotes a more effective distribution of active sites, resulting in a true synergistic effect. When Co is present in excess, this balance is disrupted: Co begins to block Pt active sites, reduces cooperative electronic effects, and may favor leaching or the formation of less active phases, ultimately decreasing catalytic activity.

**Figure 5:**
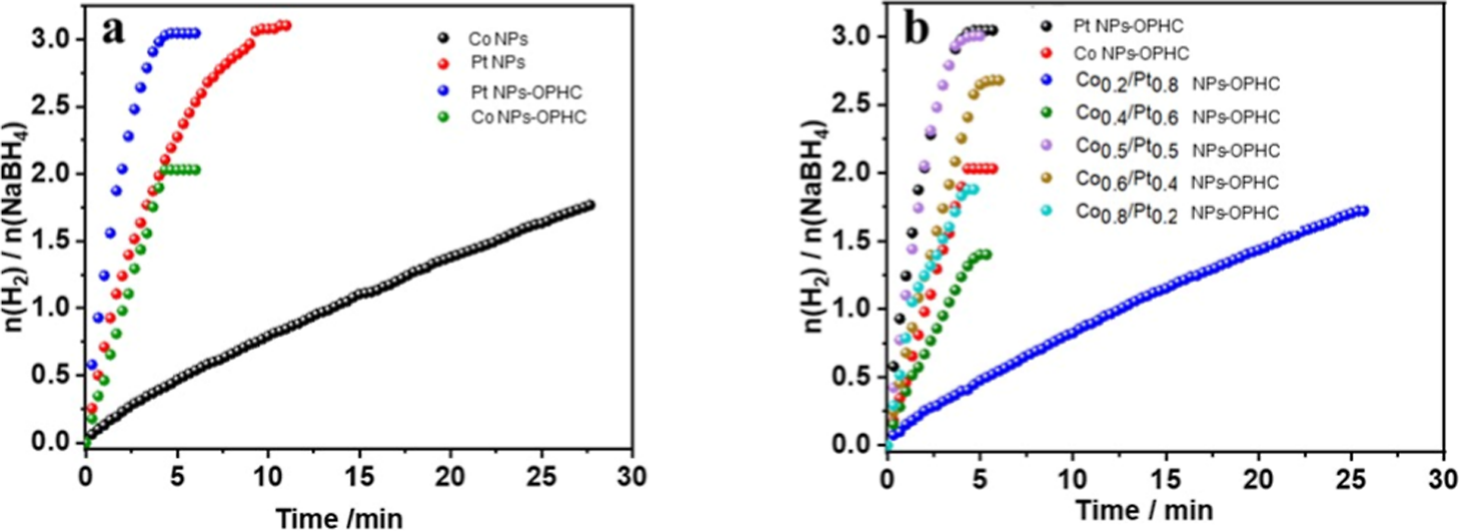
Evolution of H_2_ from NaBH_4_ hydrolysis catalyzed by Pt- and Co-based nanoparticles. (a) Comparison of unsupported Pt and Co nanoparticles (Pt NPs and Co NPs) with Pt and Co nanoparticles supported on orange-peel-derived hydrochar (Pt NPs-OPHC and Co NPs-OPHC). (b) Hydrogen evolution using Co/Pt bimetallic nanoparticles supported on orange-peel-derived hydrochar (Co/Pt NPs-OPHC) with different Co/Pt molar ratios. Experimental conditions: OPHC: 10 mg; 1.00 mL of NaBH_4_ (0.05 mol L^–1^) at 297.9 K), nanoparticle dose 0.055 mmol %.

#### 3.2.1 Evaluation of Sodium Hydroxide Effect

In general, the addition of NaOH favors NaBH_4_ hydrolysis because the alkaline medium stabilizes BH_4_^–^ and accelerates its activation on the catalytic surface, increasing H_2_ release.^41^ Therefore, the effect of adding different NaOH concentrations was evaluated. According to the results, increasing the NaOH concentration from 0.010 to 0.050 mmol L^–1^ accelerated the reaction, whereas higher concentrations (0.075 mmol L^–1^) did not provide any significant improvement and even slightly reduced the yield. This behavior occurs because moderate OH^–^ levels help remove B­(OH)_4_^–^ from active sites, keeping the catalyst available, while excess OH^–^ can compete for these sites and increase the viscosity of the medium, limiting the reaction. It is also noted that the NaOH concentration range was carefully selected to avoid significant changes in solution viscosity, ensuring that the observed effects are primarily governed by surface chemical interactions.

#### 3.2.2 Variation of Sodium Borohydride Concentration

The influence of different NaBH_4_ concentrations was evaluated in the presence of the Co/Pt NPs-OPHC catalyst, as shown in Figure 6a. It is observed that increasing the NaBH_4_ concentration accelerates the hydrogen release kinetics, reflecting the greater availability of BH_4_^–^ for interaction with the catalytic surface. Based on experimental data, a plot of ln k versus ln [NaBH_4_] was constructed (insert in Figure 6a). The linear relationship obtained indicates that the reaction mediated by Co/Pt NPs-OPHC follows fractional-order kinetics (order = 1.38) with respect to NaBH_4_ (*R* ^2^ = 0.887), the observed behavior agrees with data previously reported in the literature, such as that reported by dos Reis et al. (2024),^42^ who found a reaction order of 1.13 when using Pt/Ni bimetallic nanoparticles supported on KNbWO6 in the hydrolysis of NaBH_4_.^42^ Thus, the partial rate law (*r*) can be expressed by eq 5.

5 $$r=k{\left[{NaBH}_{4}\right]}^{1.3845}$$

**Figure 6:**
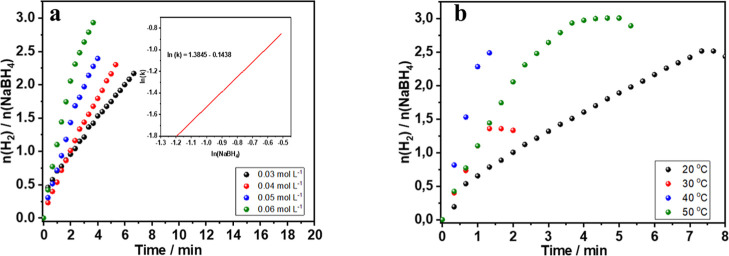
Evaluation of the effect of (a) NaBH_4_ concentration and (b) temperature on H_2_ evolution mediated by Co/Pt nanoparticles supported on orange-peel-derived hydrochar (Co/Pt NPs-OPHC).

Considering that temperature has a strong influence on the kinetics of NaBH_4_ hydrolysis, this effect was investigated. Figure 6b shows the H_2_ production using the Co/Pt NPs-OPHC catalyst at 20 °C, 30 °C, and 40 °C. It is observed that increasing the temperature significantly accelerates the reaction, resulting in higher hydrogen evolution rates. This behavior is expected, as higher temperatures increase the kinetic energy of the molecules, promoting more effective collisions between NaBH_4_ and the active sites of the catalyst, lowering the energy barrier of the process and accelerating the cleavage of B–H bonds. However, the faster hydrolysis kinetics can also favor the rapid formation of sodium metaborate, which can be strongly adsorbed onto the catalyst surfac, partially blocking active sites and decreasing catalytic efficiency.^43^

#### 3.2.3 Durability of the Catalyst

Figure 7 presents the durability assessment of the catalyst supported on orange-peel-derived hydrochar. Since long-term stability is a critical requirement for practical applications, ten consecutive NaBH_4_ hydrolysis cycles were performed under identical conditions using the Co/Pt NPs–OPHC catalyst. After 10 cycles, the catalyst retained approximately 95% of its initial activity, demonstrating excellent recyclability and structural resilience.

**Figure 7:**
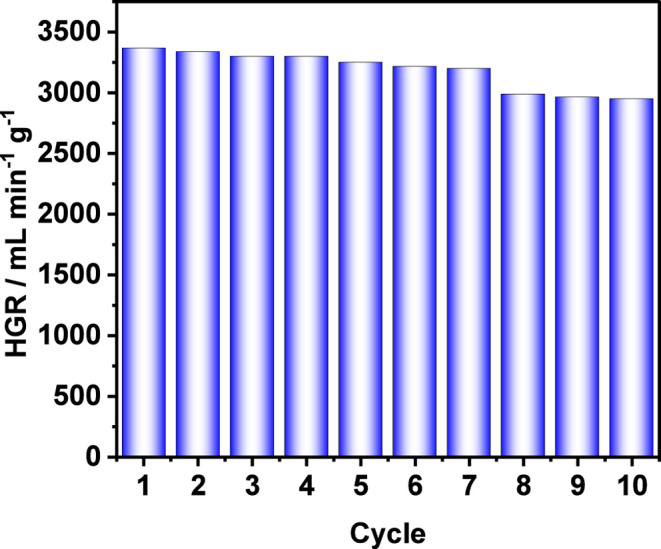
Catalyst durability in H_2_ evolution under repeated NaBH_4_ hydrolysis cycles using Co/Pt nanoparticles supported on orange-peel-derived hydrochar (OPHC). Conditions: 10 mg of OPHC; 0.055 mmol % Co/Pt NPs; 1.00 mL NaBH_4_ 0.05 mol L^–1^; 297.9 K.

The material after use was analyzed by SEM–EDS, and the results are shown in Figure S5. The presence of C, O, Pt, and Co can be observed, indicating that the active phases remained stable after the catalytic cycles. Although precipitation occurred, the material remained stable. However, the absence of quantitative leaching analyses and more detailed structural characterizations after use limits a definitive assessment of the catalyst’s chemical and morphological stability. The detection of B and Na is attributed to sodium metaborate formed during NaBH_4_ hydrolysis. The results obtained in this work were compared with previously reported literature data and are shown in Table 2.

**Table 2:** Comparison of the Activation Energy (Ea) and Hydrogen Generation Rate (HGR) Values for Hydrogen Evolution Using Cobalt-Based Catalysts Reported in the Literature with the Results Obtained in This Work

catalyst	temperature (°C)	Ea (kJ mol^–1^)	HGRs (mL min^–1^ g^–1^)	ref
3D hollow microrod engineeredMOF-derived Cu/Co	room temperature (∼25 °C)	38.66	423.4	Deonikar et al.^44^
coronavirus-like core–shell- Structured Co@C	30 °C	41.5	330	Su et al.^45^
Zn-MOF-74-derived graphene nanosheets supporting CoB alloys	30 °C	38.8	7937	Zhang et al.^35^
MOF-templated void-engineered shaggy cobalt oxide	30 °C	28.9	1571.4	Tuan et al.^46^
Co–Ru@MOF	60 °C	41.41	15,144	Onat et al.^47^
MOF-derived carbon supported cobalt catalysts	30 °C	35.09	4900	Lin et al.^48^
Co@ZIF-5	room temperature	14.95	1351	Hou et al.^49^
Co nanoflowers/rGO	30	28,1	4251,1	Wang et al.^50^
Pt/Co NPs embedded on orange peel	room temperature (∼25 °C)	41.90	3359.24	this work

#### 3.2.4 Kinetic Isotope Effect (KIE)

The Kinetic Isotope Effect (KIE) was evaluated for the Co/Pt NPs-OPHC, and a KIE value of 4.97 was obtained (Figure 8). This value falls within the typical range reported for primary isotope effects (2–7) and indicates a strong involvement of hydrogen transfer in the rate-determining step.^28^ The high KIE suggests that the activation of water molecules is the rate-limiting step in the reaction, consistent with previous studies on hydrogen transfer processes.^51^ Coelho et al.^52^ reported a KIE value of 4.36 for the Niobium Metal–Organic Framework catalyst Nb-MOF/Pt_0.4_-Co_0.6_ in the hydrolysis of NaBH_4_ for H_2_ evolution.

**Figure 8:**
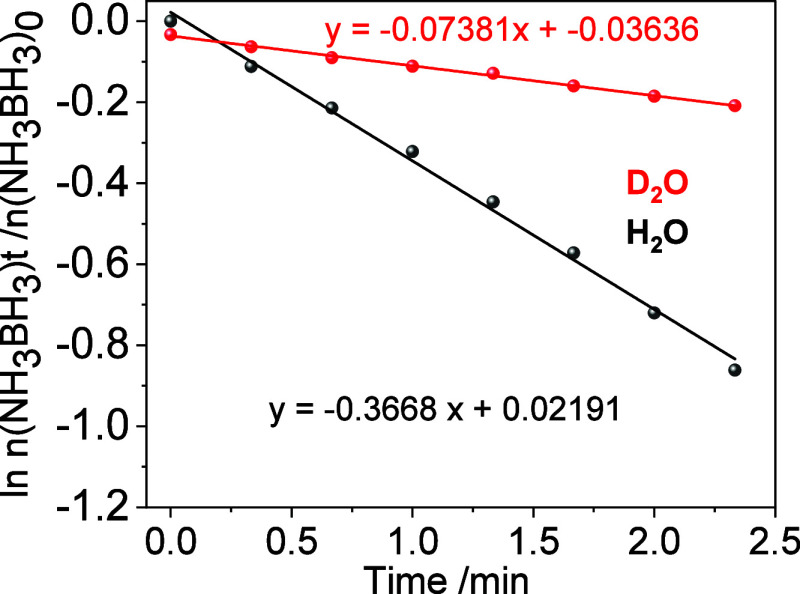
Hydrogen evolution from NaBH_4_ hydrolysis in H_2_O and D_2_O to evaluate the kinetic isotope effect (KIE). Reaction conditions: 10 mg of catalyst; 0.055 mmol % Co/Pt NPs; 1.00 mL of NaBH_4_ (0.05 mol L^–1^); 297.9 K.

Therefore, a possible mechanism for NaBH_4_ hydrolysis on the Co/Pt surface involves adsorption of borohydride species onto Co active sites, while adjacent Pt sites promote dissociative adsorption of water, generating surface H* and OH* species. The adsorbed BH_4_^–^ then reacts with activated hydrogen from water, releasing one H_2_ molecule and forming BH_3_(OH)^−^ adsorbed on the surface. This intermediate consecutively undergoes three additional hydrolysis steps, releasing three more H_2_ molecules and yielding B­(OH)_4_^–^ as the final byproduct.^53^ Thus, Co favors borohydride activation, whereas Pt enhances water dissociation, explaining the synergistic catalytic effect.

## 4 Conclusion

Orange-peel residues from essential oil extraction were successfully converted into a hydrochar support for Co/Pt nanoparticles, yielding an efficient catalyst for hydrogen evolution from NaBH_4_ hydrolysis. The hydrothermal carbonization produced a mesoporous hydrochar with abundant functional groups, promoting excellent nanoparticle dispersion and strong metal–support interactions. The Co/Pt NPs-OPHC catalyst exhibited enhanced hydrogen generation rates, with the 50:50 bimetallic composition achieving the best performance due to synergistic effects. The catalyst also demonstrated remarkable durability, retaining ∼95% activity after ten consecutive cycles. Additionally, the high kinetic isotope effect (KIE = 4.97) confirmed water activation as the rate-determining step. This work shown a sustainable strategy for valorizing agro-industrial residues into functional nanocatalysts for clean hydrogen production.

## Supplementary Material



## Data Availability

All data supporting the findings of this study are included in the manuscript and the Supporting Information.

## Notes

Additional information is available free of charge at: TGA and dTG thermograms of (a) OPR/HCl and (b) OPHC; surface properties analysis of the support (OPHC) and the catalyst (Co/PtNPs-OPHC); EDS spectra of OPHC; and EDS spectra of Co/PtNPs-OPHC (PDF)
