# Supplementary material for: Hydrochar Produced from Orange Peel Essential Oil Extraction Residues as a Support for Nanoparticles in Hydrogen Evolution Catalysis from NaBH4
Source: ACS Omega. 2026 Jul 22;11(30):44841–52. doi: 10.1021/acsomega.6c01972 (PMC13448917; doi:10.1021/acsomega.6c01972)
Supplement: Supplementary file 1 [file ao6c01972_si_001.pdf]

Supporting Information

Hydrochar Produced from Orange Peel Essential Oil Extraction Residues as a Support for Nanoparticles in Hydrogen Evolution Catalysis from $NaBH_4$

Nelson Jonathan,[1]** Renata P. L. Moreira,[2]** Noemí C. S. de Souza,[2] Amanda C. Filgueiras,[2] Garbas A. dos S. Junior,[2] Patrícia F. Pinheiro.*[2]

[1]Department of chemistry, Adamawa State college of education Hong, Adamawa State, Nigeria, Postal Code (area): 640102

[2]Departamento de Química, Universidade Federal de Viçosa, Av. Peter Henry Rolfs, s/n Campus Universitário, CEP 36570-900, Viçosa – MG, Brazil.

The authors (**) contributed equally. *Author to whom correspondence should be addressed

*Corresponding authors. E-mail addresses: patricia.pinheiro@ufv.br (Patrícia F. Pinheiro). Telephone: (55) 031 36126640.

Summary

Figure S1. TGA and dTG thermograms of (a) OPR/HCl and (b) OPHC…………………...**S1**

Table S1. Surface properties analysis of orange peel residue (OPR), orange peel residue (OPR) was treated in a dilute hydrochloric acid (HCl) solution (OPR/HCl), support (OPHC), and catalyst (Co/PtNPs-OPHC)………..**S2**

Figure S2. Scanning electron microscopy (SEM) images (a) orange peel residue (OPR) and (b) orange peel residue (OPR) was treated in a dilute hydrochloric acid (HCl) solution (OPR/HCl)…………………………………………………………………………………**S3**

Figure S3. EDS spectra of OPHC…………………………………………………….……**S4**

Figure S4. EDS spectra of Co/PtNPs-OPHC…………………………………………...….**S4**

Figure S5. SEM–EDS Chemical Mapping of Co/PtNPs-OPHC after Multiple Catalytic Cycles……………………………………..………………………………………… S5

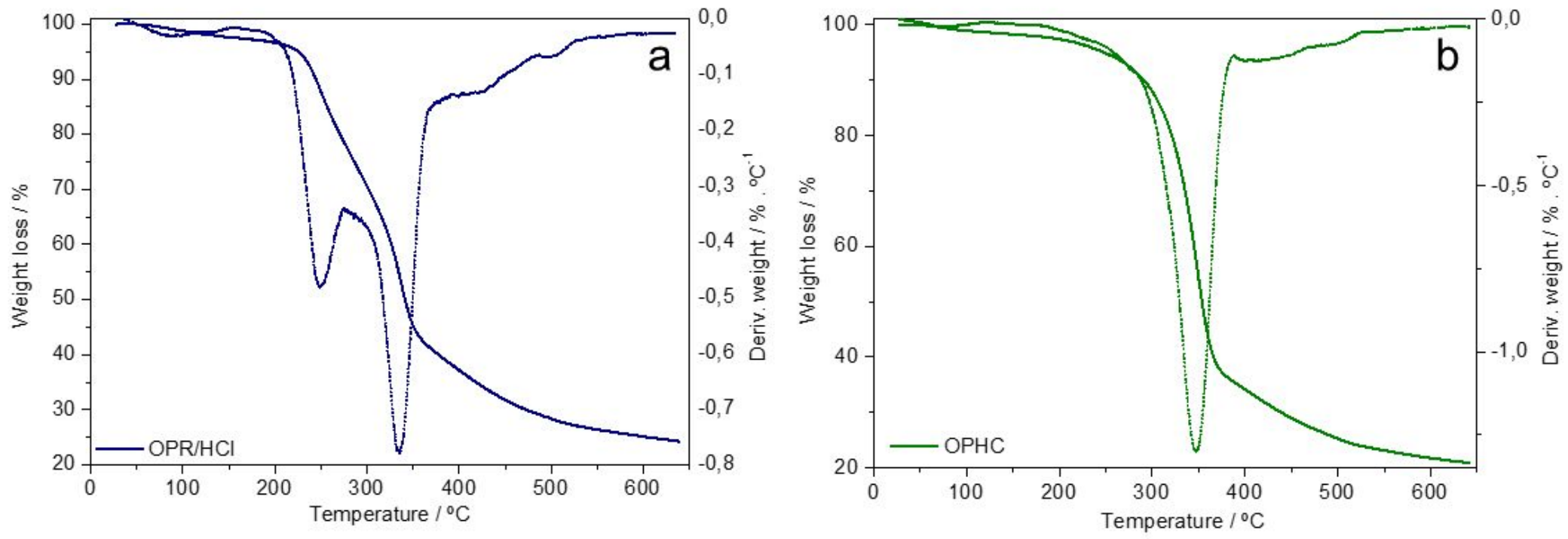

Figure S1. TGA and dTG thermograms of (a) OPR/HCl and (b) OPHC.

Table S1. Surface properties analysis of orange peel residue (OPR), orange peel residue (OPR) was treated in a dilute hydrochloric acid (HCl) solution (OPR/HCl), support (OPHC), and catalyst (Co/PtNPs-OPHC).

	Specific superface area ($m^2.g^{-1}$)	Pore radius (nm)	Pore volume (10^{-3} $cm^3.g^{-1}$)
OPR	0.97	1.54	0.98
OPR/HCl	0.00	15.96	0.27
OPHC	0.40	1.70	0.27
Co/PtNPs-OPHC	2.67	6.35	12.58

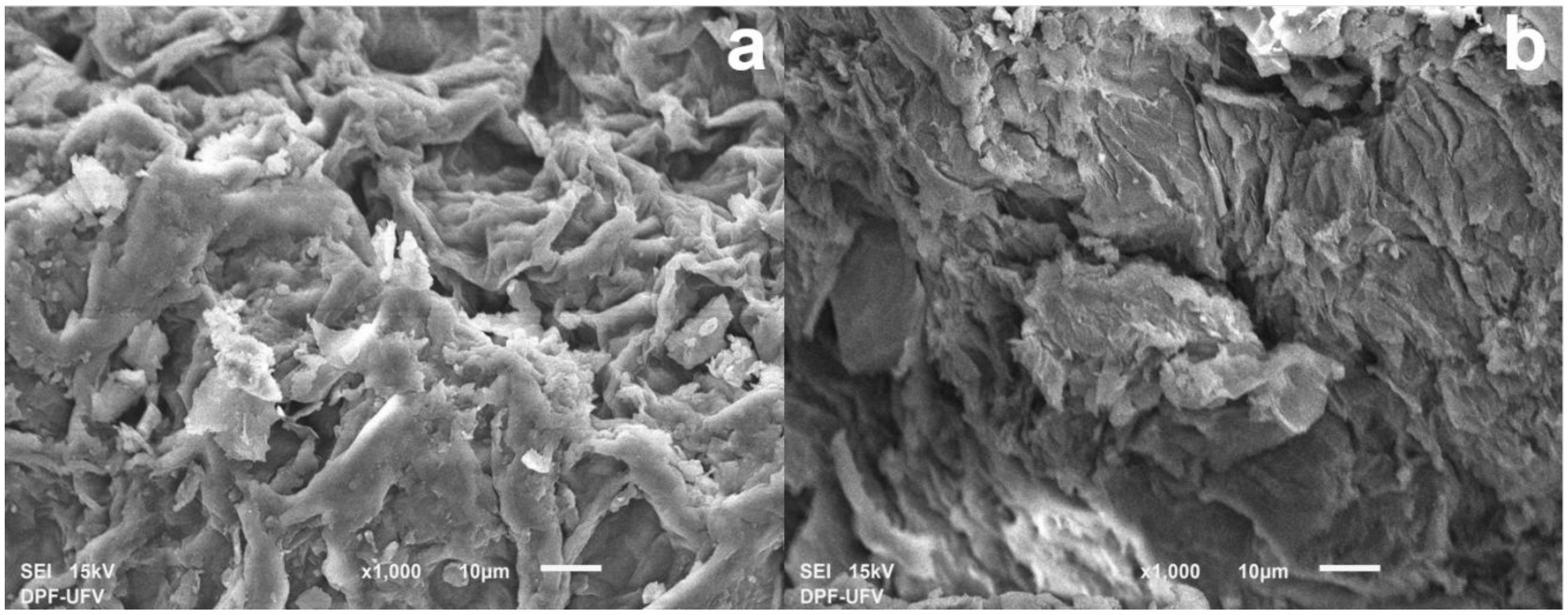

Figure S2. Scanning electron microscopy (SEM) images (a) orange peel residue (OPR) and (b) orange peel residue (OPR) was treated in a dilute hydrochloric acid (HCl) solution (OPR/HCl).

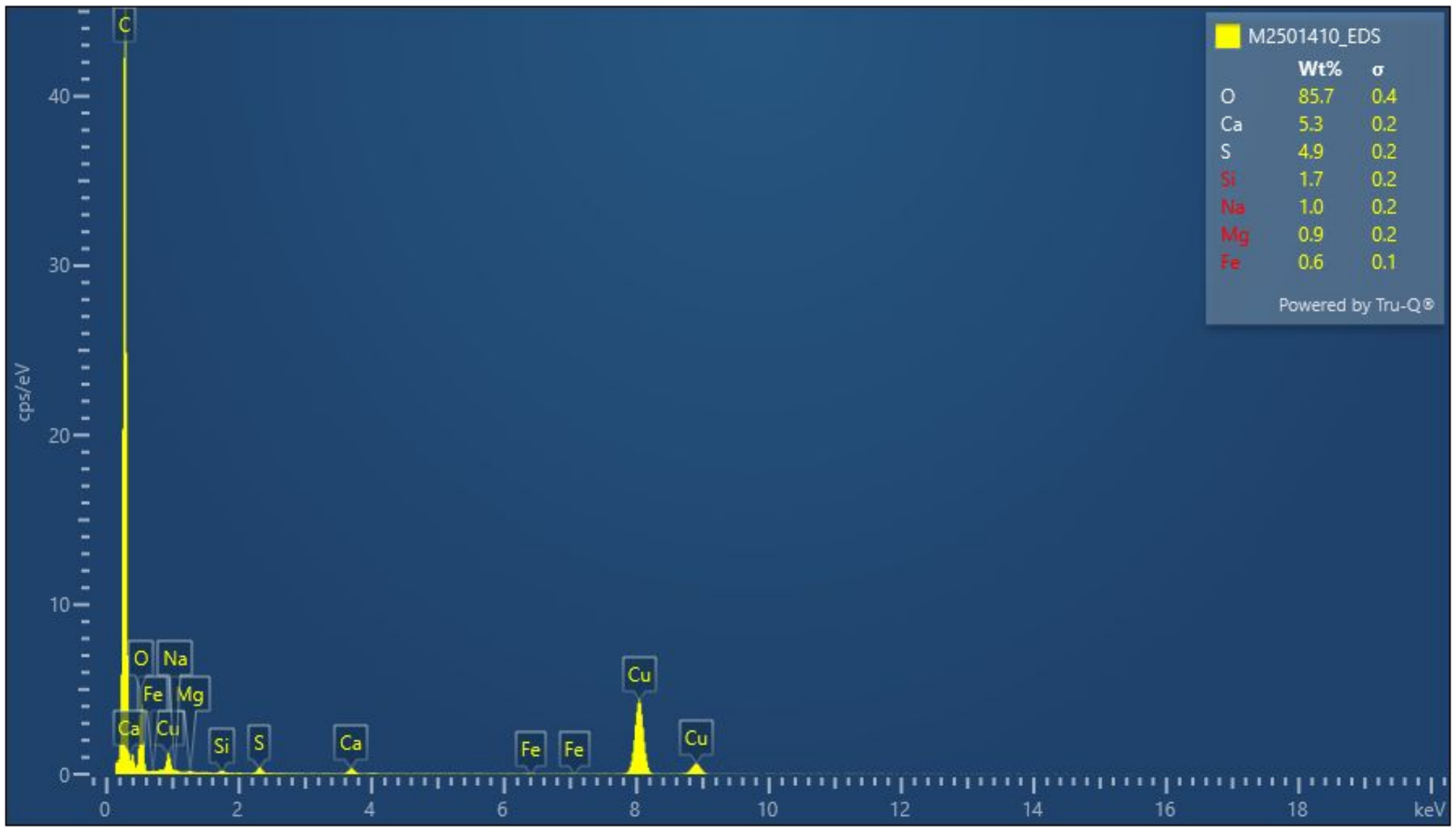

Figure S3. EDS spectra of OPHC.

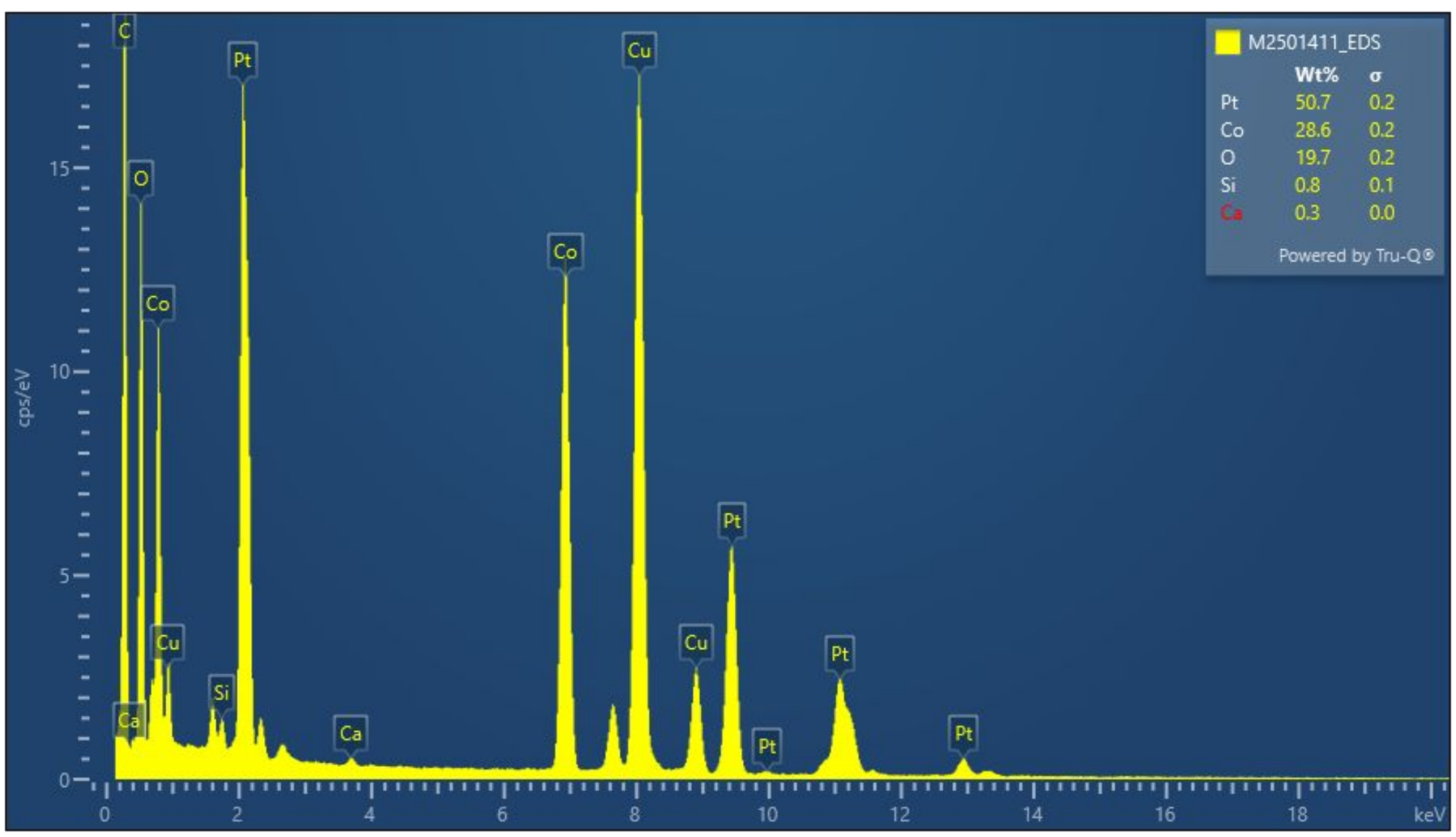

Figure S4. EDS spectra of Co/PtNPs-OPHC.

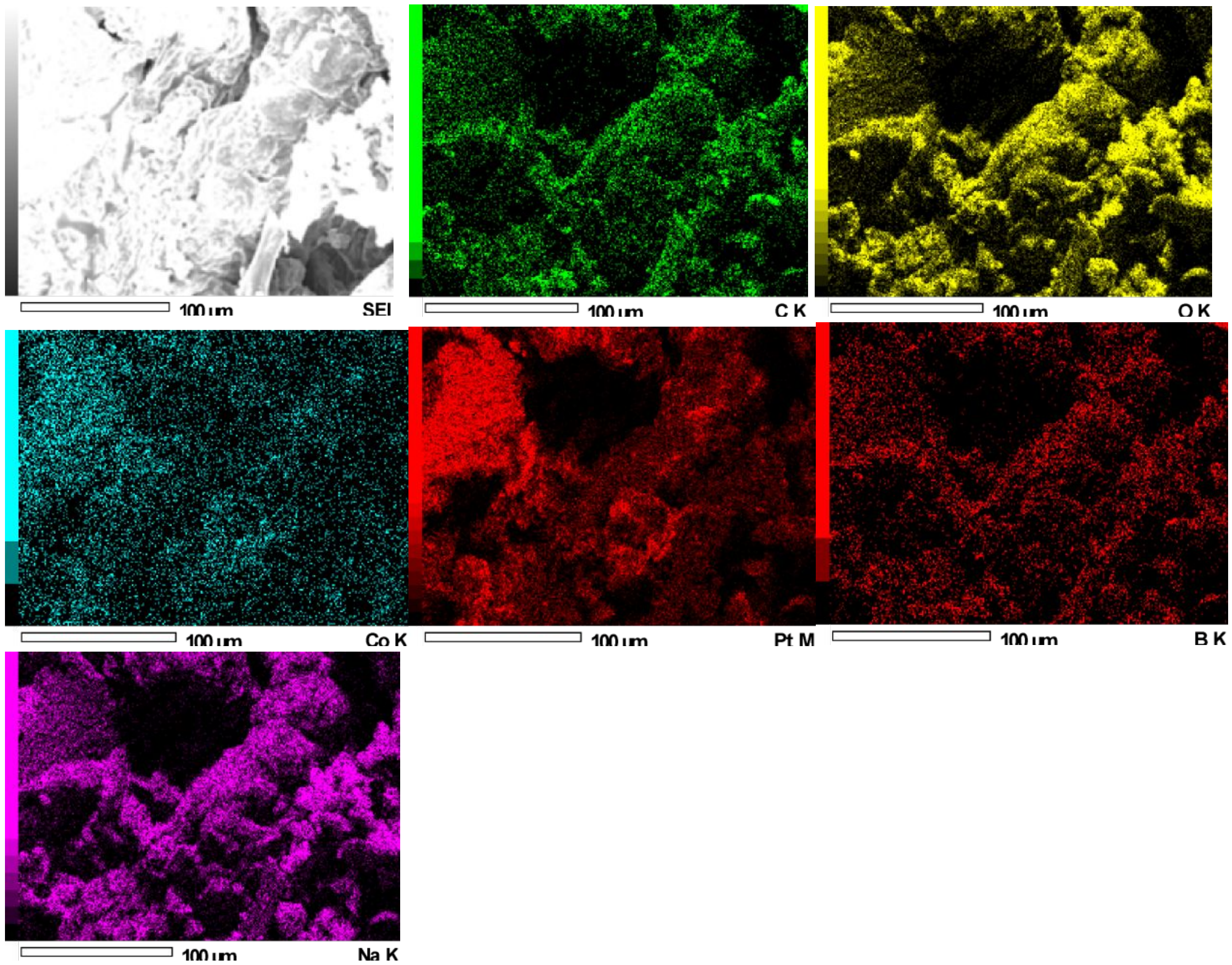

Figure S5. SEM–EDS Chemical Mapping of Co/PtNPs-OPHC after Multiple Catalytic Cycles
